# CROSS-COUNTRY COMPARISONS OF FUNCTIONING AND CARDIOVASCULAR RISKS IN OLDER ADULTS

**DOI:** 10.1093/geroni/igad104.2500

**Published:** 2023-12-21

**Authors:** Mutian Zhang, Yuan Zhang, Jennifer Ailshire, Eileen Crimmins

**Affiliations:** University of Southern California, Los Angele, California, United States; Columbia University, New York City, New York, United States; University of Southern California, Los Angeles, California, United States; University of Southern California, Los Angeles, California, United States

## Abstract

Life expectancy at birth in China has increased more than 20 years over the past 50 years and is one year higher than in the US in 2021. Whether the rapid improvement in Chinese life expectancy has been accompanied by similar changes in other aspects of the aging process compared to the US remains unclear. In this study, we focus on measured physical functioning and cardiovascular risk factors which signify disability and disease processes, and address how age and gender differences and change with age in these indicators are similar for the two countries. We use data from the 2015 China Health and Retirement Longitudinal Study (n=8,303) and the 2016/18 Health and Retirement Study (n=10,804) on performance-based functioning (e.g., walking speed) and cardiovascular biomarkers (e.g., systolic blood pressure) for those over age 60. We first examine the distribution of each measure by gender and age and conduct t-tests on mean differences. Then we conducted regression analyses to examine cross-country differences. In both countries functioning worsens with age (e.g., mean scores of walking speed for CHARLS females decreased from 0.76 to 0.47 m/s). In contrast, only among older Chinese does cardiovascular risk increase with age while in the US risk is flat (e.g., mean scores of systolic blood pressure for CHARLS females increased from 129.59 to 142.39 mm hg). Although life expectancy is nearly equivalent in these two countries, there are large gaps in health and functioning between China and the US, especially in cardiovascular disease risk.

